# Examining the effect of adverse weather on road transportation using weather and traffic sensors

**DOI:** 10.1371/journal.pone.0205409

**Published:** 2018-10-16

**Authors:** Yichuan Peng, Yuming Jiang, Jian Lu, Yajie Zou

**Affiliations:** Key Laboratory of Road and Traffic Engineering of the Ministry of Education, Tongji University, Shanghai, P.R. China; Universitat de Valencia, SPAIN

## Abstract

Adverse weather related to reduced visibility caused by fog and rain can seriously affect the mobility and safety of drivers. It is meaningful to develop effective intelligent transportation system (ITS) strategies to mitigate the negative effects of these different types of adverse weather related to reduced visibility by investigating the effect of rain and fog on traffic parameters. A number of previous researches focused on analyzing the effect of adverse weather related to reduced visibility by using simulated traffic and weather data. There are few researchers that addressed the impact of adverse weather instances using real-time data. Moreover, this paper conducts comprehensive investigation to clearly compare the changes of driving behavior and traffic parameters in adverse weather including fog and rain using real-time traffic and weather data collected by advanced vehicle-based traffic sensors and weather sensors. After some preliminary analysis, the analysis of variance method (ANOVA) was applied to further compare the significance of effects of these two kinds of adverse weather on traffic parameters. The conditional regression models were employed finally to explore the relationship between these two types of adverse weather and traffic parameters. The results would be beneficial to develop effective intelligent traffic control countermeasures under these different types of adverse weather conditions related to reduced visibility.

## 1. Introduction

Fog and rain are two types of the most common adverse weather that will lead to reduced visibility. For the purpose of reducing adverse weather related traffic crashes, at least eighteen states have installed adverse weather detection systems that reduced visibility was able to be detected in the US. However, there are still many traffic crashes occurred on the roads because of reduced visibility caused by adverse weather including fog and rain. The reduced visibility caused by different types of adverse weather also has a different impact on traffic parameters. It is then necessary to figure out the specific effect of reduced visibility caused by different types of adverse weather to minimize the adverse effect on traffic flow. It will be also very beneficial to predict and minimize the traffic crash risks of these reduced visibility events caused by adverse weather. The department of transportation will be able to provide accurate warning messages to drivers in advance. Most previous researches focus on analyzing the effect of adverse weather by using simulated traffic and weather data because real time data is hard to collect. Therefore, the major objective of this research is to examine the changes of driving behavior and traffic parameters in adverse weather conditions including fog and rain using real-time traffic and weather data collected by advanced traffic and weather sensors. The results would be helpful to develop advanced ITS strategies such as variable speed limits and dynamic advisory message in different types of adverse weather. This paper is divided into following sections. A review of existed ITS strategies including adverse weather detection and warning system and the impact of reduced visibility due to adverse weather on traffic flow and driver behaviors are provided in the next section. After that, real time traffic and weather data collection using intelligent weather and traffic sensors are introduced. The traffic parameters under different weather types are compared to investigate the effect of adverse weather on traffic parameters using analysis of variance methods and conditional regression methods. Conclusions and possible practical implementation are provided and discussed finally.

## 2. Literature review

### 2.1 Existing intelligent visibility detection and warning systems

The visibility systems applied to mitigate the negative effect of the reduction in visibility can be mainly divided into two kinds: active and passive systems [1]. Active systems mainly include driver warning systems including Lane Departure Warning system and variable speed limit signs. Passive systems comprise of various pavement markings and signs which are helpful to warn and delineate traffic [1]. By examining the configurations and management strategies of these adverse weather detection systems, it is indicated that some of these systems can accurately detect the severity of reduced visibility and respond accordingly in real time to send necessary warning messages to drivers [1]. Kilpeläinen and Summala [2] pointed out that variable message signs and web portals are efficient ways for transportation agencies to provide warning information to drivers during adverse weather conditions. All above mentioned different kinds of visibility systems should be developed based on the comprehensive understanding of the effect of reduced visibility caused by adverse weather. Shahabi et al [3] also provide a complete description of one fog detection and warning system currently active across the US. First, this system determines favorable adverse weather conditions in terms of the different meteorological components. In addition, it introduces various forecasting tools which are utilized by different agencies in their adverse weather detection processes. There are some new intelligent systems and findings that have not been included in the above-mentioned researches regarding visibility systems. The adverse weather warning system, called Adverse weather Pilot [4] collected visibility information from different sources by using advanced sensors such as satellite sensors. There is an increased use of the new visibility detection systems that are being derived from existing affordable equipment such as cameras recently because of its cost-effectiveness. It is also suggested that the utilization of airport visibility detection information might be a promising way of increasing the coverage of road visibility detection systems.

### 2.2 Effect of adverse weather on traffic flow and driving behavior

Very few researchers have compared the effect of reduced visibility due to fog and rain on the traffic parameters using field data since the vehicle based traffic data and weather data especially the reduced visibility data under fog condition are hard to collect. Most researchers have conducted driving simulator based studies in order to identify the effect of adverse weather including fog and rain separately. Broughton et al. [5] divided the fog into two levels and found that the mean headway distance in light and dense fog reduced 19 percent and 33 percent respectively compared to the sunny weather. Konstantopoulos et al. [6] analyzed drivers’eye movements in day, night and rain driving cases using driving simulator and found that visibility related factors such as driving during night and rain increase the risk of a crash. In addition, the effects of low visibility become more significant with increased experience. Yan et al. [7] also examined the influence of foggy conditions on the speed based on simulation data. They investigated the average speed in different geometric alignments and in various fog conditions. It was shown that driving speeds are significantly reduced by the existence of fog in the straight segments but the difference between speeds in light fog and fog on the straight segments was not significant. On the other hand, speeds in light adverse weather are significantly higher than that in clear conditions on S-curve segments. Some researchers found that rain will lead to the significant decrease in traffic volume [8–12]. Ghasemzadeh [13] analyzed driver lane-keeping behavior in rain using naturalistic driving data recently and found that heavy rain can significantly increase the variance of lane position. Others indicated that the severity of reduced visibility increase as the increase of precipitation. The condition of road surface is also being challenged as the precipitation increases, which will lead to the further decrease of traffic volume [14]. Muller and Trick [15] investigated the effect of driving experience on driving behavior using simulated fog data. They compared speed and hazard avoidance rates during fog between experienced drivers and novice drivers. Novice drivers exhibited higher speeds and less hazard avoidance in foggy weather. Jomaa et al. [16] evaluated the effectiveness of vehicle-activated signs on driver behavior and the trigger parameters used in each study. This research suggests that the newly-developed dynamic activation threshold values should be considered in future studies. During adverse road weather, deterioration in driving conditions reduces safe driving speeds substantially, and most drivers do not recognize those hazards, which exaggerate the crash risk. Edwards [17] proposed that adverse weather including rain and snow will deteriorate the road condition and increase the risk of traffic crashes. Recently, researchers have been trying to understand the relationship between real-time traffic parameters and crashes that occur during reduced visibility conditions. Abdel-Aty et al. [18] investigated the relationship between real-time traffic data and the risk of crashes during reduced visibility related conditions caused by adverse weather by using the data collected from loop or radar detectors and automatic vehicle identification. It was found that 73% of VR crashes could be identified. Hassan and Abdel-Aty [19] examined how real-time traffic flow data could predict crash occurrence during reduced visibility conditions. It was also shown that the factors leading to visibility-related crashes were different from those crashes occurred in clear cases. In addition to the fixed roadside sensors, in-vehicle sensors [20–21] can be considered as another data source to explore the impact of adverse weather on traffic operations. It can be concluded from above related researches that one of the common limitations of them is that most of them relied on simulation results. Moreover, few of them compared the difference of the effect of fog and rain on traffic parameters. Therefore, there is a need for further investigation that can clearly describe the driving behavior and traffic parameter changes in adverse weather conditions including fog and rain using real traffic and weather data. The results would be helpful to develop more effective ITS strategies to mitigate the negative effects of adverse weather.

## 3. Data collection and methodology

### 3.1 Weather and traffic sensor description and installation

**Fig 1:**
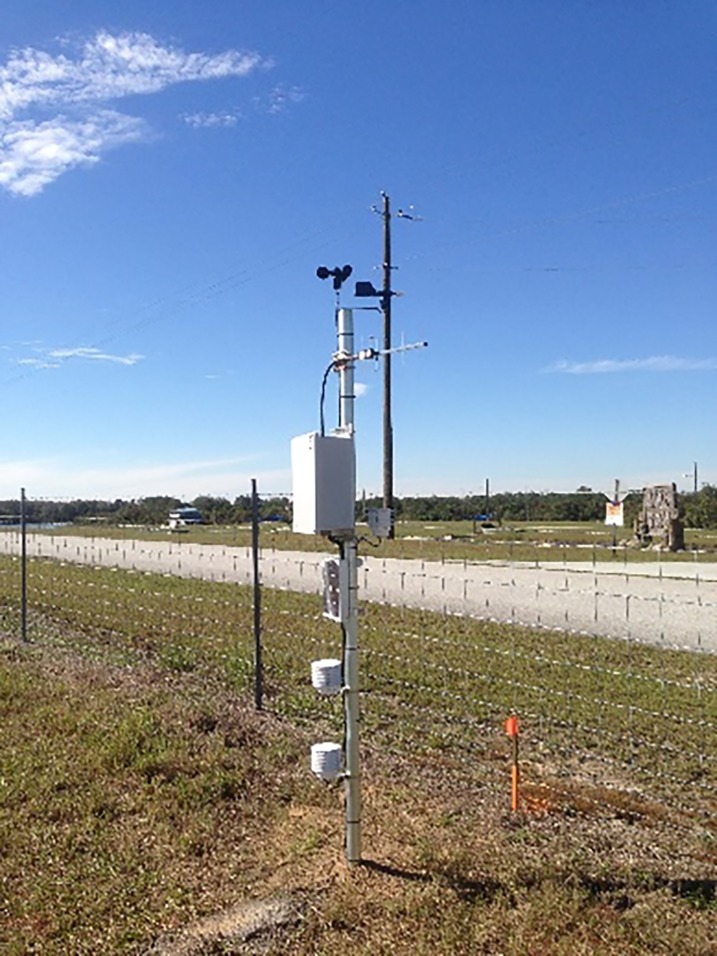
Adverse weather monitoring station.

The site selected is based on an earlier report by Abdel-Aty et al. [22] on I-4 in Polk County, where higher crashes related to adverse weather occurred. More information about the site can be referred to Peng et al. [23]. Each adverse weather monitor system (ADMS) was installed roadside and consists of three sensors at increasing elevations beginning at one foot one inch. A soil probe which is able to detect soil moisture was installed underground and an anemometer which is able to detect low wind speed was placed above the ground. A photograph of one of the ADMS is shown in Fig 1 below. The visibility sensors and an array of meteorological sensor array were installed at the center point of the ADMS to collect visibility data and other important weather parameters including precipitation. The cameras were installed to validate the data. The weather radar data being integrated into the visualization module is of high quality and reliability, updating every 5 minutes. The frequency is exactly the same as the radars used to collect weather data in this research. The adverse weather monitor system has been setup in the lab and are collecting data to ensure all sensors and communications between nodes are working appropriately before deployment in field. Data is being sent to a WSN receiver which is connected to the Internet.

**Fig 2:**
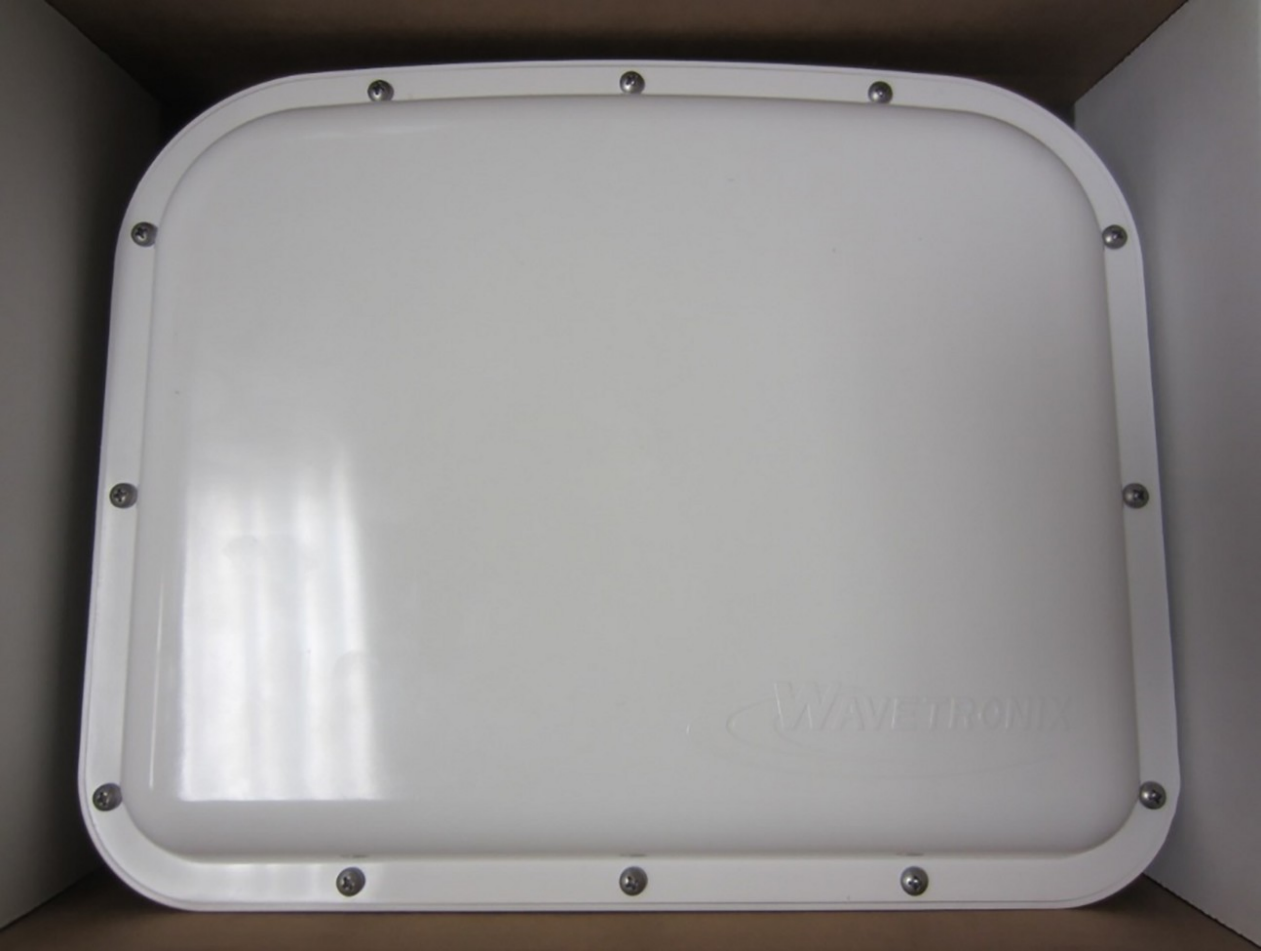
Wavetronix SmartSensor HD.

The advanced vehicle-based detector, Wavetronix SmartSensor HD(WX), was installed to collect accurate traffic flow data including vehicle speed, classification of vehicle, vehicle length and lane assignment. It was composed of a digital wave radar and the Event Logger. The range of the detection is up to 250 feet and this sensor has the ability to detect more than twenty traffic lanes at the same time. The data collected by the sensor is accurate in different traffic status. The photograph of the traffic sensor is shown in the following Fig 2. Ma et al. [24–25] applied neural network methods to analyze similar kinds of traffic data collected by remote microwave sensors. The major advantage of our sensor used in this paper is that it has the ability to detect the speeds of all the vehicles in different lanes at the same time and the measurements are very accurate.

### 3.2 Data collection

The weather and traffic data was then collected from those ADMS and WX on I-4. The weather dataset consists of twenty-one weather parameters including visibility, humidity, wind speed, wind direction, subsurface moisture and rainfall. The most important two variables used in this paper are rainfall and visibility. These two variables were combined to identify the foggy cases and rainy cases. The first variable rainfall was used to identify whether it was raining or not at that specific time on the selected site. This variable recorded the cumulative rainfall. The weather was identified as raining once the value of this variable increased. On the other hand, the other variable visibility was used to identify the fog. The weather was identified as fog once the value of visibility reduced while the value of rainfall stays the same.

The accurate vehicle-based traffic data was collected by above mentioned device. Table 1 shows a sample of the weather dataset and Table 2 shows a sample of vehicle based traffic dataset. Each observation in Table 1 provided the information about the weather on the site at the specific time and each observation provided the related information about one vehicle in Table 2.

**Table 1:** Sample of weather dataset.

Date	Time	Air Temp	Humidity	Barometric Pressure	Wind Direction	Wind Speed	Rainfall	Visibility	Subsurface Moisture
		(°F)	(%)	(Kpa)	(&)	(mph)	(inches)	(m)	(VWC)
2015/5/29	0:00:01				N	0			0
2015/5/29	0:00:27				ENE	0			0.1654
2015/5/29	0:00:36				NNE	0			0
2015/5/29	0:01:36				ENE	0			0
2015/5/29	0:04:00	70	80	30.1	NE	1.3	0.56		
2015/5/29	0:04:29				ENE	0			0.232
2015/5/29	0:05:44				N	0			0
2015/5/29	0:06:46				NNE	0			0
2015/5/29	0:07:16				ENE	0			0.1654
2015/5/29	0:07:47				N	0			0
2015/5/29	0:08:57				N	0			0
2015/5/29	0:09:01	70	81	30.1	ENE	0	0.56		
2015/5/29	0:10:05				ENE	0			0.232
2015/5/29	0:10:08							2000	

**Table 2:** Sample of vehicle-based traffic dataset.

Date	Time	Lane	Speed(mph)	Length(ft)	Range(ft)	Class	Duration (0.001s)
3/14/2015	14:51.2	2	72	23.2	160.1	1	286
3/14/2015	14:51.5	4	70.3	215	162.4	1	260
3/14/2015	14:51.8	2	63.4	24.5	84	2	302
3/14/2015	14:52.2	1	65.1	24.7	174.1	1	303
3/14/2015	14:52.4	3	72.3	23.9	160.1	1	275
3/14/2015	14:52.7	1	67.2	19.1	72	2	261
3/14/2015	14:53.2	4	67.5	18	173.1	1	225
3/14/2015	14:54.2	5	71.4	20.5	158.1	1	252
3/14/2015	14:55.1	5	74.2	23.2	86	2	283
3/14/2015	14:56.2	2	72.7	24.5	120.6	2	291

The above mentioned two types of original datasets were then merged into one combined dataset using two common variables date and time to analyze the effect of adverse weather on traffic parameters.

### 3.3 Methodology description

Analysis of variance method was employed at first to compare the effect of different types of adverse weather including fog and rain on traffic parameters. Matched case-control logistic regression was then adopted in this paper to further explore the relationship between adverse weather and traffic parameters. This model was ever employed in few transportation studies on traffic safety [26]. It is assumed that there are N strata with one low visibility case and m clear cases with good visibility in stratum j, where j = 1, 2, 3 …… N. The probability of any observation in a stratum being a foggy case or a rainy case was modeled by the following linear logistic regression model:

$$\log it\{P_{j}(X_{ij})\}=\alpha_{j}+\beta_{1}X_{1ij}+\beta_{2}X_{2ij}+\ldots .+\beta_{k}X_{kij}$$ (1)

Where Pj (X_ij_) is the probability that the ith observation in the jth stratum being a low visibility case; X_ij_ = (X_1ij_, X_2ij_, …….. X_kij_) is the vector of k traffic flow variables; i = 0, 1, 2 …….m and j = 0, 1, 2 …N.

## 4. Comparison analysis

### 4.1 Preliminary comparison of speed

**Fig 3:**
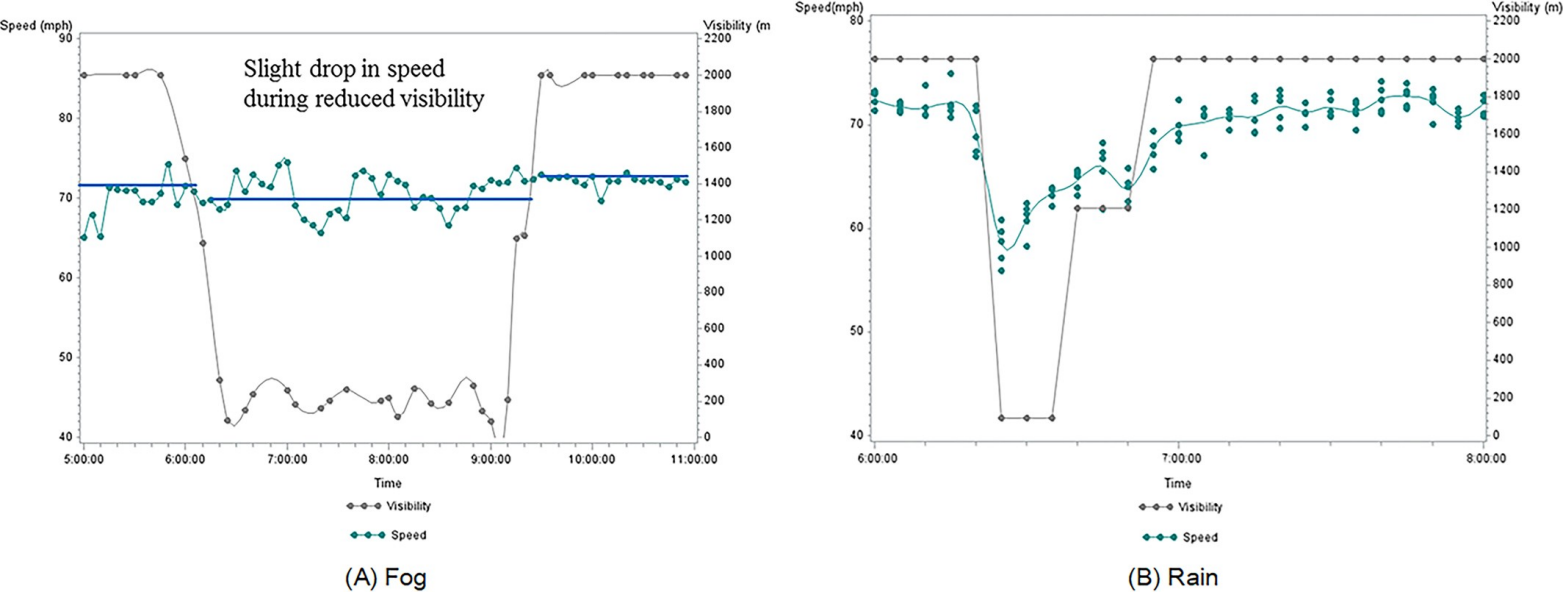
Comparison of speed and visibility during fog and rain. (A) Fog (B) Rain.

A typical foggy case and rainy case was selected to compare the different effects on the traffic parameters. The period of foggy case is from 6:30am to 9:00am in the morning. The relationship between mean speed and visibility was shown on the left side of the following Fig 3. It can be seen from this figure that there is a slight drop in speed during reduced visibility. The mean speed drops to around 70 mph during the fog period. Accordingly, a rainy case was selected at the similar time as fog in order to compare both better. The period of rain was from 6:30am to 7:00am. The relationship between mean speed and visibility is shown on the right side of the following Fig 3. It can be seen from this figure that there is a significant drop in speed at the beginning of reduced visibility caused by rain. The mean speed is lower as the visibility decreases. The mean speed drops to below 60 mph at the beginning of the rain when the visibility is only around 200m and goes back to around 70 mph after the rain. The visibility reduced in both cases and the impact of reduced visibility on speed is more significant during rain compared to fog.

### 4.2 Comparison results using ANOVA

For the purpose of comparing the effect of fog and rain on traffic parameters in further, this paper applied ANOVA method to compare the differences of four important traffic parameters including speed, standard deviation of speed, headway and standard deviation of headway. It is noted that this research applied several Q-Q plots to check distribution of each variable before applying ANOVA method and found that each variable follows the normal distribution and there is not significant difference in variance. After that, the ANOVA method was employed to further analyze the effects of reduced visibility caused by rain and fog on different types of vehicles and different lanes.

The following Table 3 shows the comparison of four traffic parameters by dividing the whole dataset into three different cases: clear cases when the visibility is greater than or equal to 2000 m, foggy cases when the visibility is less than 2000m but without any rainfall and rainy case. The period of one minute was considered as a sample of clear case, foggy case or rainy case. The sample size of clear case, foggy case and rainy case is 13465, 1873, and 3697, respectively. It can be seen from Table 3 that speed would decrease significantly as the weather type changed from clear case to foggy case or rainy case. The mean speed reduced more significantly during the rainy case compared to the weather type changed from clear case to foggy case, which further convinced the results of above preliminary analysis. Drivers are more willing to reduce their speeds when it rains compared to the foggy case. The mean speed of all the vehicles reduced 12.36 kilometers per hour (kph) during the rain. On the other hand, the mean headway and the standard deviation of headway increased significantly as the weather type changed from clear case to foggy case or rainy case. The mean headway and the standard deviation of headway increased more significantly during the rainy case compared to the foggy case, which means drivers are more willing to keep distance with the leading vehicles during the rain. The standard deviation of speed is higher in fog and rain but the result is not significant.

**Table 3:** Comparison of traffic parameters for all the vehicles.

All vehicles	Traffic parameters			
Weather types	Speed(kph)	Headway (s)	Standard deviation of speed(kph)	Standard deviation of headway(s)
clear-fog	4.88*	-0.24*	-0.71	-3.81*
fog-rain	7.48*	-1.29*	-0.16	-0.11
clear-rain	12.36*	-1.53*	-0.87	-3.92*

* means the difference is significant in 0.05 level.

All the vehicles were further divided into passenger cars and trucks for the purpose of figuring out whether the impact of these two weather types on traffic parameters is different for different vehicle types. The length of thirty feet was considered as the border line for the classification according to previous research [19]. Table 4 and Table 5 show the summary for the results of comparison. Similar as above analysis, the duration of one minute was considered as a clear case or adverse weather case. The mean speed, headway, standard deviation of speed and headway during each period was calculated. Therefore, the sample size of clear case, foggy case and rainy case is the same as above analysis.

**Table 4:** Comparison of traffic parameters for passenger cars.

Passenger cars	Traffic parameters			
Weather types	Speed(kph)	Headway (s)	Standard deviation of speed(kph)	Standard deviation of headway(s)
clear-fog	4.96*	-0.28*	-0.87*	-3.96*
fog-rain	7.57*	-1.32*	-0.26	-0.24
clear-rain	12.53*	-1.60*	-1.13*	-4.20*

* means the difference is significant

**Table 5:** Comparison of traffic parameters for trucks.

Trucks	Traffic parameters			
Weather types	Speed(kph)	Headway (s)	Standard deviation of speed(kph)	Standard deviation of headway(s)
clear-fog	3.24*	-0.16*	-0.32	-3.42*
fog-rain	6.01*	-1.17*	-0.14	-0.06
clear-rain	9.25*	-1.33*	-0.46	-3.48*

* means the difference is significant in 0.05 level

It can be seen from the Table 4 and Table 5 that some results are similar as the results shown in Table 3. The mean values of speed, headway and standard deviation of headway are significantly different between the clear cases and adverse weather cases including fog and rain for both vehicle types. The mean speed reduced more significantly during the rainy case compared to the weather type changed from clear case to foggy case for both vehicle types. The mean headway and the standard deviation of headway increased more significantly during the rainy case compared to the foggy case for both vehicle types.

Specifically, the mean speed decreases 4.96 kph for passenger cars while it decreases only 3.24kph for trucks during the foggy cases. In addition, the mean speed decreases 12.53 kph for passenger cars while it decreases only 9.25kph for trucks during the rainy cases. The value of mean headway increases 0.28s for passenger cars while it increases only 0.16s for trucks and the value of standard deviation of headway increases 3.96s for passenger cars while it increases only 3.42s for trucks during the foggy cases. In comparison, the value of mean headway increases 1.60s for passenger cars while it increases only 1.33s for trucks and the value of standard deviation of headway increases 4.20s for passenger cars while it increases only 3.48s for trucks during the rainy cases. In addition, the effect of fog and rain on standard deviation of speed of trucks is not significant while the standard deviation of speed of passenger cars significantly increases by 0.87 kph and 1.13kph on average during the fog and rainy cases separately. Therefore, it can be concluded from the results that the impact of adverse weather on passenger cars is more significant compared to trucks.

Moreover, the effects of adverse weather fog and rain on different lanes were further analyzed in this section. The datasets used in this section was similar as above analysis, the duration of one minute was considered as a clear case or adverse weather case. The mean speed, headway, standard deviation of speed and headway of all the vehicles in each lane during each period was calculated. The sample size of clear case, foggy case and rainy case is still the same as above analysis.

The speed and headway comparison of the outer lane that is close to the roadside under different weather types is shown in Table 6. It was indicated from the table that the mean speeds in rainy cases and foggy cases are both significantly lower than mean speed in clear cases. The difference of mean speed between foggy cases and clear cases is lower than the difference between rainy cases and clear cases. The difference of mean speed between clear case and foggy case is 4.67 kph and the difference between clear case and rainy case is 12.01 kph. The difference of mean headway between clear case and foggy case is 0.18s and the difference between clear case and rainy case is 1.37s. The effect of adverse weather on speed and headway becomes more significant in rainy cases compared to the foggy cases for the outer lane. The standard deviation of headway in fog and rain is higher compared to those in clear cases but the difference of standard deviation of headway in fog and rain is not significant. The standard deviation of speed in fog and rain is higher although it is not significant about the difference.

**Table 6:** Comparison of traffic parameters for different outer lanes.

Outer lane	Traffic parameters			
Weather types	Speed(kph)	Headway (s)	Standard deviation of speed(kph)	Standard deviation of headway(s)
clear-fog	4.67*	-0.18*	-0.51	-3.67*
fog-rain	7.34*	-1.19*	-0.09	-0.02
clear-rain	12.01*	-1.37*	-0.60	-3.69*

* means the difference is significant in 0.05 level.

The comparison of traffic parameters on the middle lane under different weather types is shown in Table 7. It was also indicated from the table that the mean speeds in rainy cases and foggy cases are both significantly lower than mean speed in clear cases. The difference of mean speed between foggy cases and clear cases is lower than the difference between rainy cases and clear cases. The difference of mean speed between clear case and foggy case is 4.84 kph and the difference between clear case and rainy case is 12.28 kph. Both values are larger than those in the outer lane, which means the effect of adverse weather on speed is more significant in the middle lane compared to the outer lane. The difference of mean headway between clear case and foggy case is 0.21s and the difference between clear case and rainy case is 1.49s. Both values are also larger than those in the outer lane, which means the effect of adverse weather on headway is more significant in the middle lane compared to the outer lane. The effect of adverse weather on speed and headway becomes more significant in rainy cases compared to the foggy cases for the middle lane. The standard deviation of headway in fog and rain is higher compared to those in clear cases. The standard deviation of speed in rain is significantly higher than in clear cases.

**Table 7:** Comparison of traffic parameters for different middle lanes.

Middle Lane	Traffic parameters			
Weather types	Speed(kph)	Headway (s)	Standard deviation of speed(kph)	Standard deviation of headway(s)
clear-fog	4.84*	-0.21*	-0.64	-3.76*
fog-rain	7.44*	-1.28*	-0.14	-0.08*
clear-rain	12.28*	-1.49*	-0.78*	-3.84*

* means the difference is significant in 0.05 level.

The speed and headway comparison of the inner lane that is close to the median of the road segment under different weather types is shown in Table 8. It also can be seen from the table that the mean speeds in rainy cases and foggy cases are both significantly lower than mean speed in clear cases. The difference of mean speed between foggy cases and clear cases is lower than the difference between rainy cases and clear cases. The difference of mean speed between clear case and foggy case is 4.97 kph and the difference between clear case and rainy case is 12.58 kph. Both values are larger than those in the middle lane, which means the effect of adverse weather on speed is the most significant in the inner lane compared to the middle lane and outer lane. The difference of mean headway between clear case and foggy case is 0.37s and the difference between clear case and rainy case is 1.71s. Both values are also larger than those in the middle lane, which also indicates the effect of adverse weather on headway is the most significant in the inner lane. For the inner lane, it is shown that both the standard deviation of headway and the standard deviation of speed in fog and rain is significantly higher than those in clear cases.

**Table 8:** Comparison of traffic parameters for different inner lanes.

Inner Lane	Traffic parameters			
Weather types	Speed(kph)	Headway (s)	Standard deviation of speed(kph)	Standard deviation of headway(s)
clear-fog	4.97*	-0.37*	-0.81*	-3.97*
fog-rain	7.61*	-1.34*	-0.24	-0.24*
clear-rain	12.58*	-1.71*	-1.05*	-4.21*

* means the difference is significant in 0.05 level.

## 5. Modeling results

The conditional logistic regression model was applied in this study to figure out the relationship between three different weather types and traffic flow characteristics in further. The mainly advantage of this model compared to those logistic models applied in random non-matching sampling is that it has the ability to minimize the effect of other factors that the investigator wishes to avoid. At first, samplings of clear cases are selected and some non-traffic flow variables associated with each case of adverse weather including fog and rain are selected as matching factors. The variables used to match cases and controls are: day of the week and time of adverse weather occurs. The data of all corresponding adverse weather cases including foggy cases and rainy cases were extracted from the combined dataset and a total of three times of observations with clear case were randomly selected from the combined dataset.

The following four traffic parameters: mean speed and headway, variance of speed and headway were the major objective of this modeling investigation and were then used as input in the model. It is noted that the all these four kinds of traffic parameters were calculated based on the vehicle based traffic data in one minute. The weather type was considered as dependent variable in this model and was divided into three types to investigate the relationship between traffic flow characteristics and adverse weather. The weather type was considered as 0 for the clear case when the visibility is greater than or equal to 2000m and the value of the variable rainfall stays the same. The weather type was classified as 1 for the foggy case when the value of visibility reduced while the value of the variable rainfall stays the same and the weather type was classified as 2 for the rainy case when there is an increase of the rainfall variable. The modeling result was shown in the following Table 9:

**Table 9:** Modeling results for three weather types.

Parameter	DF	Parameter Estimate	Standard Error	Chi-Square	Pr > ChiSq	Hazard Ratio
**speed**	1	-0. 458	0.012	14.658	< .0001	0.787
**Speed standard deviation**	1	0.031	0.004	8.369	0.008	1.007
**headway**	1	0.406	0.095	15.236	< .0001	1.236
**Headway standard deviation**	1	0.213	0.081	12.257	< .0001	1.104
**Model Fit Statistics**						
**Criterion**			**Without covariates**		**With covariates**	
**-2Log Likelihood**			**1286.32**		**1218.68**	
**AIC**			**1286.32**		**1226.68**	
**BIC**			**1286.32**		**1238.73**	

The modeling results further confirmed our previous results in above sections, which indicated that increase of mean of headway, variance of speed and headway were related to the weather change from clear weather to the adverse weather while the decrease of mean speed was related to the weather change from clear weather to the adverse weather. In addition, the change of mean speed is the most significantly compared to the other variables when the weather changes. It is also shown from the model fit statistics that the parameter estimates by applying conditional logistic regression had more precision compared to logistic models without matching because of lower AIC and BIC.

## 6. Conclusions and discussions

This paper applied ANOVA and conditional regression method to analyze the weather data and vehicle-based traffic data collected by advanced traffic and weather sensors. This paper investigated the effect of adverse weather on four important traffic parameters: speed, standard deviation of speed, headway and standard deviation of headway. There are several major conclusions summarized from this study.

The visibility reduced in both foggy and rainy cases and the impact of adverse weather on speed is more significant during rain compared to fog. The mean speed would decrease significantly as the weather type changed from clear case to foggy case or rainy case. Drivers are more willing to reduce their speeds when it rains compared to the foggy case. On the other hand, the mean headway and the standard deviation of headway increased significantly as the weather type changed from clear case to foggy case or rainy case. The mean headway and the standard deviation of headway increased more significantly during the rainy case compared to the foggy case, which means drivers are more willing to keep distance with the leading vehicles during the rain. In addition, it can be concluded from the results that the impact of adverse weather on passenger cars is more significant compared to trucks since the difference of all the four traffic parameters are larger for cars compared to trucks when the weather changes from clear cases to fog or rain. The effect of adverse weather on headway is the most significant in the inner lane. It is shown in the inner lane that both the standard deviation of headway and the standard deviation of speed in fog and rain is significantly higher than those in clear cases. The conditional modeling results further confirmed the results that increase of mean of headway, variance of speed and headway were related to the weather change from clear weather to the adverse weather while the decrease of mean speed was related to the weather change from clear weather to the adverse weather. In addition, the change of mean speed is the most significantly compared to the other variables when the weather changes. Overall, the effect of rain on all these four traffic parameters are more significant than fog, especially on mean speed and headway.

With the development of technology that has been made in traffic and weather data collection and real-time communication area, it is plausible to detect and predict adverse weather case areas in real time. Real-time measurements of traffic parameters and weather can help in warning drivers when the reduced visibility related to fog has fallen below certain acceptable levels or the level of rain has increased to certain unsafe levels. The credibility of detection about change of weather and traffic pattern is essential to ensure drivers’ compliance with these warning systems. Moreover, how drivers react to the effect of weather change is crucial to the effectiveness of adverse weather warning systems. This study comprehensively investigated the relationship between traffic and adverse weather based on field data collected by advanced traffic and weather sensors. Most of previous related researches focused on analysis based on simulated data. The results will be beneficial to understand how different types of adverse weather affect traffic and also how changes in traffic can indicate to traffic management centers that there is a weather problem. In addition, the result is helpful for researchers in the future to develop more accurate and reliable adverse weather warning message or more accurate dynamic advisory message to mitigate the negative effect of adverse weather. The traffic management centers will be able to send warning messages in the future when they detect certain special traffic patterns caused by certain types of adverse weather and it would be beneficial to reduce related congestion or crashes caused by adverse weather. For future works, the results of this research can be further verified and compared with data collected in other districts. It is also important to investigate the fog-related highway accident-prone spots [27] and develop some variable speed limit strategies for adverse weather based on the optimization algorithms [28].

## 7. Limitations of the study

As it was pointed out at the end of the conclusion and discuss section, the current conclusions of this paper are mainly based on the data collected on the highway I-4 in Polk County, Florida. Additional description about the data can be referred to Peng et al.[23]. It is meaningful to collect the traffic and weather data from other types of road segments to obtain more comprehensive analysis results on the effect of adverse weather in the future. In addition, this research mainly focused on analyzing the macroscopic impact of adverse weather on traffic parameters by using statistical methods including ANOVA and conditional logistic modeling. The microscopic impact of adverse weather on each specific vehicle can also be investigated to develop weather-type dependent car-following models.
